# Association of Restrictive Housing During Incarceration With Mortality After Release

**DOI:** 10.1001/jamanetworkopen.2019.12516

**Published:** 2019-10-04

**Authors:** Lauren Brinkley-Rubinstein, Josie Sivaraman, David L. Rosen, David H. Cloud, Gary Junker, Scott Proescholdbell, Meghan E. Shanahan, Shabbar I. Ranapurwala

**Affiliations:** 1 Center for Health Equity Research, University of North Carolina at Chapel Hill; 2 Department of Social Medicine, University of North Carolina at Chapel Hill; 3 Injury Prevention Center, University of North Carolina at Chapel Hill; 4 Division of Infectious Diseases, University of North Carolina at Chapel Hill; 5 Department of Behavioral Sciences and Health Education, Emory University, Atlanta, Georgia; 6 North Carolina Department of Public Safety, Raleigh; 7 North Carolina Department of Public Health, Raleigh; 8 Department of Epidemiology, University of North Carolina at Chapel Hill

## Abstract

**Importance:**

Restrictive housing, otherwise known as solitary confinement, during incarceration is associated with poor health outcomes.

**Objective:**

To characterize the association of restrictive housing with reincarceration and mortality after release.

**Design, Setting, and Participants:**

This retrospective cohort study included 229 274 individuals who were incarcerated and released from the North Carolina prison system from January 2000 to December 2015. Incarceration data were matched with death records from January 2000 to December 2016. Covariates included age, number of prior incarcerations, type of conviction, mental health treatment recommended or received, number of days served in the most recent sentence, sex, and race. Data analysis was conducted from August 2018 to May 2019.

**Exposures:**

Restrictive housing during incarceration.

**Main Outcomes and Measures:**

Mortality (all-cause, opioid overdose, homicide, and suicide) and reincarceration.

**Results:**

From 2000 to 2015, 229 274 people (197 656 [86.2%] men; 92 677 [40.4%] white individuals; median [interquartile range (IQR)] age, 32 years [26-42]), were released 398 158 times from the state prison system in North Carolina. Those who spent time in restrictive housing had a median (IQR) age of 30 (24-38) years and a median (IQR) sentence length of 382 (180-1010) days; 84 272 (90.3%) were men, and 59 482 (63.7%) were nonwhite individuals. During 130 551 of 387 913 incarcerations (33.7%) people were placed in restrictive housing. Compared with individuals who were incarcerated and not placed in restrictive housing, those who spent any time in restrictive housing were more likely to die in the first year after release (hazard ratio [HR], 1.24; 95% CI 1.12-1.38), especially from suicide (HR, 1.78; 95% CI, 1.19-2.67) and homicide (HR, 1.54; 95% CI, 1.24-1.91). They were also more likely to die of an opioid overdose in the first 2 weeks after release (HR, 2.27; 95% CI, 1.16-4.43) and to become reincarcerated (HR, 2.16; 95% CI, 1.99-2.34).

**Conclusions and Relevance:**

This study suggests that exposure to restrictive housing is associated with an increased risk of death during community reentry. These findings are important in the context of ongoing debates about the harms of restrictive housing, indicating a need to find alternatives to its use and flagging restrictive housing as an important risk factor during community reentry.

## Key Points

**Question:**

Is restrictive housing, otherwise known as solitary confinement, during incarceration associated with an increased risk of mortality after release into the community?

**Findings:**

This cohort study included 229 274 people who were released from incarceration in North Carolina from 2000 to 2015. Compared with individuals who were incarcerated and not placed in restrictive housing, individuals who spent any time in restrictive housing were 24% more likely to die in the first year after release, especially from suicide (78% more likely) and homicide (54% more likely); they were also 127% more likely to die of an opioid overdose in the first 2 weeks after release.

**Meaning:**

The results of this study suggest that exposure to restrictive housing as a condition of confinement is associated with an increased risk of death during community reentry.

## Teaser

This cohort study evaluates whether restrictive housing, otherwise known as solitary confinement, during incarceration is associated with reincarceration and mortality after release.

## Introduction

Risk of death after incarceration is high.^1,2^ A study in Washington found that, in the first 2 weeks after release, the risk of death among those who had been recently incarcerated was 12.7-fold that of their nonincarcerated counterparts. Similarly, a recent North Carolina study found that, 1 year after release from prison, people who were recently incarcerated were 40 times as likely to die of an opioid overdose as their nonincarcerated peers.^3^ Social and economic instability attributable to poor access to housing, employment, and health care likely contribute to increased risk of death during community reentry.^4,5^ However, while the associations of incarceration with instability and mortality after release have been drawn, little is known about how the conditions of confinement during incarceration may be associated with mortality risk.

*Restrictive housing*, more commonly referred to as *solitary confinement* or *segregation*, is defined as the practice of isolating individuals who are incarcerated in small cells for 22 to 24 hours a day. People housed in these settings are exposed to social isolation, sensory deprivation, and physical idleness. Additionally, these individuals have less access to programming, visitation, and other privileges available to the general population. Correctional systems typically use restrictive housing for disciplinary purposes (eg, when someone breaks a rule) or for administrative purposes (eg, to isolate someone who may otherwise be at risk of experiencing or committing violence). In 2017, data from 43 prison systems accounting for 81% of the imprisoned population in the United States demonstrated that an average of approximately 4.5% of people who were incarcerated were also in restrictive housing.^6^ Of these 43 prison systems, 30 tracked length of stay, reporting that most individuals spent less than a year in restrictive housing settings; however, 25 systems reported that more than 3500 people were held for more than 3 years, 67% of whom had been in restrictive housing for more than 6 years.^6^ In 2015, the United Nations revised the Standard Minimum Rules on the Treatment of Prisoners to include the Mandela Rules, which for the first time clearly defined restrictive housing and provided guidelines on its use.^7^ Specifically, the rules call for an end to prolonged restrictive housing, defined as a period of more than 14 days.^7^

Correctional systems rely on restrictive housing as a punishment for violating prison rules and as a security measure, claiming that it provides protection and safety for those who may be unsafe if housed with the general incarcerated population. However, reliance on restrictive housing is not without risks. A growing body of literature has documented the association of restrictive housing with the health of people who have been incarcerated.^8,9,10,11^ Individuals with mental illness are overrepresented in most restrictive housing units. These individuals are particularly susceptible to psychological deterioration while isolated in restrictive housing, which can manifest as reclusiveness, social withdrawal, psychosis, self-harm, posttraumatic stress disorder, and suicide.^12,13,14^ However, to our knowledge, no study has examined the association of restrictive housing with mortality after release. We address this gap with the current study by examining how restrictive housing was associated with mortality after release, including all-cause mortality, opioid overdose death, homicide, and suicide, and reincarceration in North Carolina between 2000 and 2016. In addition, we examined the association of mortality with the following: (1) repeated restrictive housing stays, (2) time spent in restrictive housing using the Mandela Rules guidelines, and (3) the effect measure modification in these associations by race, given the racial disparities in incarceration rates in the United States.

## Methods

### Study Design and Population

We conducted a retrospective cohort study to assess the association of restrictive housing with mortality after release among individuals who were incarcerated in the North Carolina state prison system from January 1, 2000, to December 31, 2015. Prison release data were obtained from the North Carolina Department of Public Safety (NCDPS). The NCDPS data were linked to publicly available North Carolina death records from January 1, 2000, to December 31, 2016. Person-time was calculated from the day of release from prison until death, reincarceration, or the end of 2016. Individuals who remained incarcerated throughout the study period, who died prior to release (n = 59), or who died on the day of release (n = 959) did not contribute person-time and were therefore excluded from the study. This is because, in most cases, when a death occurs in prison, the individual’s record is marked with a release on the same day. The linkage, accrual of person-time, and the sample have been described previously.^3^ The institutional review boards of the NCDPS and the University of North Carolina at Chapel Hill approved this study, including a waiver for informed consent because of the secondary nature of the data. All analyses were conducted between August 2018 and May 2019, and this study followed the Strengthening the Reporting of Observational Studies in Epidemiology (STROBE) reporting guideline.

### Exposure and Outcome Definitions

We considered 3 measures of restrictive housing, as follows: (1) the exposure of interest, a binary measure (ie, yes or no) of being placed in restrictive housing during an incarceration, (2) the number of restrictive housing placements during an incarceration (ie, 0, 1-2, >2), and (3) the amount of time spent in restrictive housing during an incarceration (ie, 0 days, >0 to 14 days, >14 days) based on the Mandela Rules.^7^

We examined 4 postrelease mortality outcomes, as follows: (1) all-cause death, (2) opioid overdose death, (3) homicide death, and (4) suicide death. Because reincarceration was a significant competing risk for these outcomes, we also examined its association with restrictive housing to better contextualize the results. Mortality outcomes were examined in the linked NCDPS death records data. A binary variable was created for each of the mortality outcomes. All-cause and cause-specific death diagnoses were defined using the *International Statistical Classification of Diseases and Related Health Problems, Tenth Revision *(*ICD*-*10*) codes from the North Carolina death records. An opioid overdose death was identified using *ICD*-*10* code T40.0 (opium), T40.1 (heroin), T40.2 (other opioids, commonly prescribed opioids), and T40.4 (other synthetic narcotics, commonly fentanyl or its analogs). Homicide death was identified using *ICD*-*10* codes X85 to Y09 and Y87.1. Suicide death was identified using *ICD*-*10 *codes X60 to X84 and Y87.0. All outcomes were examined at 2 weeks, 1 year, and complete follow-up after release; 1-year mortality after release was the primary outcome measure.

### Covariates

Covariate information was obtained from the NCDPS data. To identify potential covariates to control for confounding in this study, we developed a directed acyclic graph, which helped identify a minimally sufficient set of well-measured covariates that controlled for all measured confounding.^15,16^ The minimally sufficient set of covariates included time-varying age (ie, <25, 25-50, >50 years), number of prior incarcerations (ie, 0, 1-2, >2), drug-related convictions (ie, yes or no), violence-related convictions (ie, yes or no), mental health treatment recommended (ie, yes or no), mental health treatment received (ie, yes or no), quartiles of number of days served in the most recent sentence (ie, <87, 87-177, 178-399, >399), and time-fixed sex (ie, male or female) and race (ie, white or nonwhite). Drug-related conviction and violence-related conviction variables were developed using cause of incarceration codes within the NCDPS data.

### Statistical Analysis

We used Cox proportional hazard regression to examine the association of restrictive housing with mortality after release, with the Lin, Wei, and Weissfeld^17^ robust variance estimator to account for person-level clustering. To adjust for time-varying confounding, we used inverse probability of exposure weighted marginal structural models.^18,19,20^ The inverse probability weights for restrictive housing were calculated as follows: w = *P* of restrictive housing / *P* of restrictive housing | age, number of prior incarcerations, drug-related conviction, violence-related conviction, mental health recommendation and treatment, and days served, where w indicates inverse probability weight and *P* indicates probability.

The inverse probability weights were derived using logistic regression.^18,19,20^ Marginal structural Cox proportional hazard models were then weighted along with adjustment for time-fixed sex and race to estimate the adjusted hazard ratios (aHRs) and 95% CIs.^18,19,20^ Competing risks owing to reincarceration and other causes of deaths were addressed by censoring the person-time at the time of such events. An interaction term between exposure and race was added to examine race-associated estimates, and aHRs, 95% CIs, and *P *for interaction from the regression models were noted.

We conducted sensitivity analysis to examine the following: (1) the association of the percent-time spent in restrictive housing during an incarceration period with mortality after release and reincarceration and (2) the outcomes of alternative statistical adjustment with a 5-category mental health treatment recommendation based on an in-prison mental health screening inventory: no intervention; outpatient intervention with psychologist or clinical social worker; outpatient pharmacological intervention with psychiatrist, psychologist, or clinical social worker; long-term residential and pharmacological intervention with psychiatrist, psychologist, or clinical social worker; or acute inpatient intervention with psychiatrist, psychologist, or clinical social worker. All data analyses were conducted using SAS version 9.4 (SAS Inc).

## Results

Between January 1, 2000, and December 31, 2015, 229 274 people were released 398 158 times from the state prison system in North Carolina; 197 656 (86.2%) were men, 92 677 (40.4%) were white, and they had a median (interquartile range [IQR]) age of 32 years (26-42) years. However, 2094 people (0.9%) with 10 245 incarcerations (2.6%) who were missing restrictive housing information and covariate information were excluded, leaving a final sample of 387 913 releases (Table 1). More than one-third of the individuals (87 050 [38.0%]) were incarcerated multiple times (range, 2-31). Overall, the 229 274 individuals included in the study accrued 1 974 823 nonincarcerated person-years during the 16-year study period. The median (IQR) time spent in prison was 176 (86-395) days. Most people released had less than high school education (148 827 [65.2%]). Individuals screened positive for substance use disorder in 268 893 of 387 913 incarcerations (69.3%) and were recommended for mental health treatment at intake in 47 166 incarcerations (12.2%).

**Table 1.** Characteristics of Individuals Who Were Incarcerated by History of Restricted Housing Exposure in North Carolina, 2000-2016

Characteristic	No. (%)	
	No Restrictive Housing	Restrictive Housing
Persons, No.	135 943	93 331
Incarcerations, No.	257 362	130 551
**Person-Level Characteristics**^a^		
Age, median (IQR), y	34 (26-42)	30 (24-38)
Men	113 384 (83.4)	84 272 (90.3)
Race/ethnicity		
White	58 828 (43.3)	33 849 (36.3)
Nonwhite	77 115 (56.7)	59 482 (63.7)
Married^b^	36 776 (14.3)	16 431 (12.6)
Mortality	9482 (7.0)	4604 (4.9)
Opioid overdose	867 (0.6)	454 (0.5)
Homicide	702 (0.5)	759 (0.8)
Suicide	363 (0.3)	272 (0.3)
Education^c^		
Did not complete high school	82 587 (61.0)	66 240 (71.2)
High school graduate or completed GED	50 272 (37.2)	25 254 (27.1)
Some college	2437 (1.8)	1543 (1.7)
**Incarceration-Level Characteristics**^d^		
Mental health treatment recommended^e^	27 427 (10.7)	19 739 (15.1)
Mental health treatment received^f^	5927 (2.3)	13 276 (10.2)
Substance use disorder	181 870 (70.7)	87 023 (66.7)
Drug-related conviction	62 082 (24.1)	23 270 (17.8)
Violence-related conviction	39 869 (15.5)	38 268 (29.3)
Incarceration length, median (IQR), d	129 (71-238)	382 (180-1010)
Reincarceration	118 921 (45.8)	60 139 (46.1)

Abbreviations: GED, general education degree; IQR, interquartile range.

^a^ Individuals categorized into restrictive housing if they were ever in restrictive housing.

^b^ Data for 255 individuals missing.

^c^ Data for 1022 individuals missing.

^d^ Individuals categorized into restrictive housing for each incarceration.

^e^ Data for 712 incarcerations missing.

^f^ Data for 12 incarcerations missing.

### Characteristics of Those Who Were in Restrictive Housing

During 130 551 of 387 913 incarcerations (33.7%), people were placed in restrictive housing. In 59 476 incarcerations (15.3%), a person was placed in restrictive housing once, and in 71 075 incarcerations (18.3%), a person was placed in restrictive housing 2 or more times. In 89 336 restrictive housing episodes (68.4%), the duration of restrictive housing was for more than 14 days. Compared with incarceration episodes during which people were not placed in restrictive housing, episodes with restrictive housing involved individuals who were more likely to be younger (median [IQR] age, 34 [26-42] years vs 30 [24-38] years), to be male (113 384 [83.4%] vs 84 272 [90.3%]), to have had less than a high school education (82 587 [61.0%] vs 66 240 [71.2%]), to have been recommended for mental health treatment (27 427 [10.7%] vs 19 739 [15.1%]), to have received in-prison mental health treatment (5927 [2.3%] vs 13 276 [10.2%]), to have had a violence-related conviction (39 869 [15.5%] vs 38 268 [29.3%]), and to have had longer sentences (median [IQR], 129 [71-238] days vs 382 [180-1010] days). Compared with incarceration episodes without restrictive housing, incarceration episodes with restrictive housing included lower proportions of people with substance use disorders (181 870 [70.7%] vs 87 023 [66.7%]) and people with drug-related convictions (62 082 [24.1%] vs 23 270 [17.8%]) (Table 1).

### Mortality and Reincarceration Outcomes During the Study Period

A total 14 086 deaths occurred after release: 1321 (9.4%) opioid overdose deaths, 1461 (10.4%) homicide deaths, and 635 (4.5%) suicide deaths (32 opioid overdose deaths were also suicides). In addition, there were 178 032 reincarcerations from 2000 to 2016. Compared with individuals who were incarcerated and not placed in restrictive housing, those who spent time in restrictive housing were more likely to die in the first year after release of all causes (aHR, 1.24; 95% CI, 1.12-1.38), especially from suicide (aHR, 1.78; 95% CI, 1.19-2.67) and homicide (aHR, 1.54; 95% CI, 1.24-1.91) (Table 2). They were also more likely to die of an opioid overdose in the first 2 weeks after release (aHR, 2.27; 95% CI, 1.16-4.43) and to become reincarcerated (aHR, 2.16; 95% CI, 1.99-2.34) (Figure).

**Table 2.** Association of Restrictive Housing During Incarceration With 1-Year Mortality After Release and Reincarceration in North Carolina, 2000-2016

Restrictive Housing Placements	Deaths or Reincarcerations, No.	Person-Years	Adjusted Hazard Ratio (95% CI)^a^		
			Overall	Among White Individuals	Among Nonwhite Individuals
**All-Cause Mortality**					
None	1557	236 433	1 [Reference]	1 [Reference]	1 [Reference]
Any	847	117 634	1.24 (1.12-1.38)	1.29 (1.12-1.46)	1.19 (1.01-1.37)
1	365	54 280	1.17 (1.03-1.33)	1.28 (1.08-1.52)	1.05 (0.87-1.28)
≥2	482	57 162	1.38 (1.14-1.66)	1.33 (1.03-1.73)	1.42 (1.08-1.85)
**Reincarceration**					
None	36 751	236 433	1 [Reference]	1 [Reference]	1 [Reference]
Any	22 558	117 634	1.46 (1.42-1.49)	1.42 (1.37-1.47)	1.48 (1.44-1.52)
1	9163	54 280	1.22 (1.19-1.25)	1.19 (1.14-1.24)	1.24 (1.20-1.28)
≥2	13 395	57 162	1.89 (1.82-1.96)	1.83 (1.72-1.96)	1.92 (1.84-2.01)
**Opioid Overdose Deaths**					
None	227	236 433	1 [Reference]	1 [Reference]	1 [Reference]
Any	105	117 634	1.27 (0.97-1.67)	1.38 (1.03-1.83)	0.82 (0.37-1.80)
1	53	54 280	1.26 (0.91-1.74)	1.31 (0.93-1.85)	1.01 (0.41-2.45)
≥2	52	57 162	1.24 (0.77-1.98)	1.45 (0.88-23.8)^b^	0.34 (0.11-1.03)^b^
**Homicide Deaths**					
None	230	236 433	1 [Reference]	1 [Reference]	1 [Reference]
Any	207	117 634	1.54 (1.24-1.91)	1.10 (0.63-1.90)	1.67 (1.32-2.12)
1	78	54 280	1.29 (0.97-1.72)	0.82 (0.40-1.65)	1.43 (1.04-1.96)
≥2	129	57 162	1.70 (1.20-2.40)	2.04 (0.77-5.41)	1.61 (1.17-2.21)
**Suicide Deaths**					
None	86	236 433	1 [Reference]	1 [Reference]	1 [Reference]
Any	57	117 634	1.78 (1.19-2.67)	2.00 (1.26-3.15)	1.18 (0.53-2.59)
1	27	54 280	1.55 (0.95-2.50)	1.95 (1.16-3.30)^b^	0.37 (0.08-1.68)^b^
≥2	30	57 162	2.29 (0.99-5.27)	2.30 (0.79-6.71)	2.17 (0.81-5.81)

^a^ Adjusted for age, sex, race, prior incarcerations, time in incarceration, violence-related convictions, drug-related convictions, mental health screening recommendation, and mental health treatment receipt.

^b^ *P* value for interaction < .05.

**Figure.**
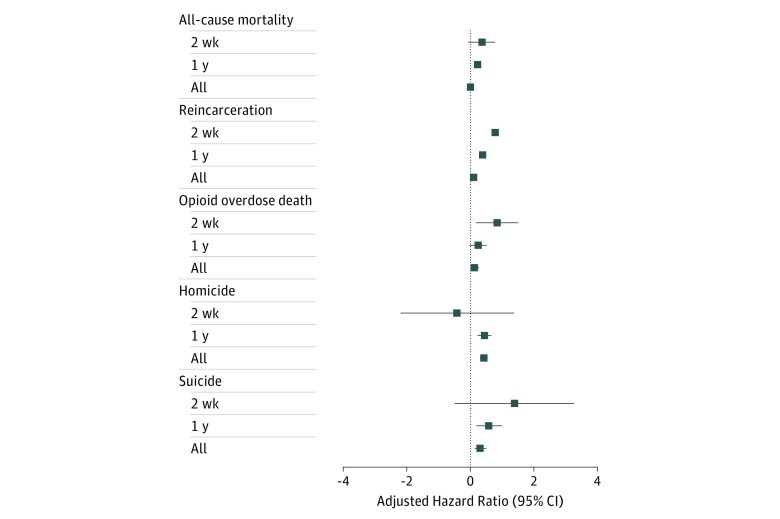
Association of Restrictive Housing During Incarceration With Mortality After Release and Reincarceration in North Carolina, 2000-2016 All hazard ratios are adjusted for sex, race, and time-varying factors including age, prior incarcerations, time in incarceration, violence-related convictions, drug-related convictions, mental health screening recommendation, and mental health treatment receipt. The x-axis is on a logarithmic scale. Whiskers represent 95% CIs.

We observed a dose-response for all-cause mortality, homicide, suicide, and reincarceration in the first year after release: compared with individuals with no restrictive housing placements, those with 2 or more restrictive housing placements had a greater risk of death or reincarceration (all-cause mortality: aHR, 1.38; 95% CI, 1.14-1.66; homicide: aHR, 1.70; 95% CI, 1.20-2.40; suicide: aHR, 2.29; 95% CI, 0.99-5.27; reincarceration: aHR, 1.89; 95% CI, 1.82-1.96). However, a dose response was not observed for opioid overdose deaths (aHR, 1.24; 95% CI, 0.77-1.98) (Table 2). Similarly, we found that those who spent more than 14 consecutive days in restrictive housing had a greater risk of all-cause mortality, homicide, suicide and reincarceration within 1 year after release but not of opioid overdose deaths than those with 0 days of restrictive housing (all-cause mortality: aHR, 1.34; 95% CI, 1.15-1.56; homicide: aHR, 1.61; 95% CI, 1.23-2.11; suicide: aHR, 1.81; 95% CI, 1.08-3.06; reincarceration: aHR, 1.72; 95% CI, 1.66-1.76; opioid overdose death: aHR, 1.24; 95% CI, 0.80-1.92) (Table 3). Further, the association of restrictive housing with opioid overdose death and suicide after release was more pronounced among white individuals compared with nonwhite individuals (opioid overdose death: aHR, 1.38 [95% CI, 1.03-1.83] vs aHR, 0.82 [95% CI, 0.37-1.80]; suicide: aHR, 2.00 [95% CI, 1.26-3.15] vs aHR, 1.18 [95% CI, 0.53-2.59]) (Table 2). The association of restrictive housing with homicide and reincarceration was higher for nonwhite individuals compared with white individuals (homicide: aHR, 1.67 [95% CI, 1.32-2.12] vs aHR, 1.10 [95% CI, 0.63-1.90]; reincarceration: aHR, 1.48 [95% CI, 1.44-1.52] vs aHR, 1.42 [95% CI, 1.37-1.47]) (Table 2).

**Table 3.** Association of Time Spent in Restrictive Housing During Incarceration With 1-Year Mortality After Release and Reincarceration in North Carolina, 2000-2016

Time Spent in Restrictive Housing, d	Deaths or Reincarcerations, No.	Person-Years	Adjusted Hazard Ratio (95% CI)^a^		
			Overall	Among White Individuals	Among Nonwhite Individuals
**All-Cause Mortality**					
0	1557	236 433	1 [Reference]	1 [Reference]	1 [Reference]
>0 to 14	269	40 365	1.17 (1.01-1.35)	1.30 (1.07-1.57)	1.04 (0.83-1.29)
>14	580	77 265	1.34 (1.15-1.56)	1.36 (1.10-1.69)	1.31 (1.07-1.62)
**Reincarceration**					
0	36 751	236 433	1 [Reference]	1 [Reference]	1 [Reference]
>0 to 14	5886	40 365	1.11 (1.08-1.15)	1.13 (1.07-1.19)	1.11 (1.06-1.15)
>14	16 671	77 265	1.72 (1.66-1.76)	1.63 (1.55-1.71)^b^	1.77 (1.71-1.83)^b^
**Opioid Overdose Deaths**					
0	227	236 433	1 [Reference]	1 [Reference]	1 [Reference]
>0 to 14	41	40 365	1.35 (0.95-1.92)	1.41 (0.98-2.05)	1.05 (0.37-2.95)
>14	64	77 265	1.24 (0.80-1.92)	1.41 (0.89-2.24)	0.50 (0.18-1.41)
**Homicide Deaths**					
0	230	236 433	1 [Reference]	1 [Reference]	1 [Reference]
>0 to 14	54	40 365	1.21 (0.87-1.66)	1.25 (0.60-2.60)	1.20 (0.84-1.72)
>14	153	77 265	1.61 (1.23-2.11)	0.82 (0.40-1.67)^b^	1.84 (1.36-2.48)^b^
**Suicide Deaths**					
0	86	236 433	1 [Reference]	1 [Reference]	1 [Reference]
>0 to 14	21	40 365	1.72 (1.01-2.94)	2.23 (1.26-3.94)	0.31 (0.04-2.31)
>14	36	77 265	1.81 (1.08-3.06)	1.90 (1.01-3.55)	1.56 (0.64-3.76)

^a^ Adjusted for age, sex, race, prior incarcerations, time in incarceration, violence-related convictions, drug-related convictions, mental health screening recommendation, and mental health treatment receipt.

^b^ *P* value for interaction < .05.

Sensitivity analyses suggested that the association of the percent-time spent in restrictive housing during incarceration with mortality after release had a dose-response relationship, such that increasing percent-time spent in restrictive housing was associated with greater mortality after release (eTable in the Supplement). Adjustment with a 5-category mental health treatment recommendation variable only changed the effect estimates at the third decimal place, suggesting that the time-varying binary mental health variables used in the main analysis produced results that were robust to confounding from mental health disorders.

## Discussion

To our knowledge, this study is the first to examine the association of restrictive housing with mortality after release. We found that people who had spent any time in restrictive housing during incarceration in a state prison in North Carolina were significantly more likely to die of all causes in the first year after release than those who did not. In addition, our results demonstrated that death by suicide and homicide in the first year and opioid overdose in the first 2 weeks after release were more common among those who had experienced restrictive housing compared with those who were incarcerated but never in restrictive housing. Further, the risk of death and reincarceration was higher among individuals with more restrictive housing placements and among those who spent more than 14 consecutive days in restrictive housing placements.

Previous research has shown that the period after release is a time of increased risk of death for all who have experienced recent incarceration.^1,3^ Our results go a step further and suggest that exposure to restrictive housing, as a condition of confinement, may be a contributing factor to the risk of death during community reentry. Our findings also point to an exacerbation of risk when people are placed in restrictive housing repeatedly and for longer periods, underscoring the importance of the Mandela Rules guidelines. However, for opioid overdose and reincarceration, any time at all in restrictive housing is associated with increased risk. We also found differences in risk by racial group that mirror known population-level mortality trends (eg, white individuals are at increased risk of opioid overdose compared with nonwhite individuals).^21,22,23^ These results can be used to identify people for linkage to trauma-informed, community-based substance use and mental health treatment, overdose prevention and harm reduction, and wraparound care and services.

In recent years, much energy has been devoted to improving the carceral environment and limiting the use of restrictive housing because of its potential negative effects on those who experience it. In 2010, the American Bar Association published guidelines for reforming the use of restrictive housing that advised against long-term restrictive housing for disciplinary purposes and advocated for the allowance of more programming and out-of-cell time and the close monitoring of mental health deterioration. Similarly, in 2016, the Department of Justice issued recommendations concluding that although the use of restrictive housing—particularly when used to ensure safety—may sometimes be necessary, it should be rare.^24^ The same year, the American Correctional Association issued similar recommendations relevant to the use of restrictive housing.^25^ Empirical studies documenting the consequences of restrictive housing on health outcomes have played a prominent role in advancing state and local reforms and advocacy. Findings from this study contribute to this body of literature.

In 2015, the NCDPS, in collaboration with the Vera Institute of Justice, began implementing important reforms in the use of restrictive housing.^26^ At this time, NCDPS prohibited the use of restrictive housing among individuals younger than 18 years, mandated staff training, and created 2 units: the Therapeutic Diversion Unit, which acts as an alternative to restrictive housing for people with severe mental illness, and the Rehabilitative Diversion Unit, which aids in transition from restrictive housing to the general prison population. These types of programs are an initial step to eliminating the harms that restrictive housing may have on health outcomes after release. Other possible interventions could include providing individuals who have experienced restrictive housing with more comprehensive, trauma-informed discharge planning services that include linkage to mental health and substance use treatment providers and increased access to stabilizing resources in the community, such as housing and employment opportunities.^27^ It also underscores the importance of overdose education and naloxone distribution programs at reentry from incarceration.

### Limitations

Despite this article’s important findings, this study has limitations. It is important to note that, given the observational study design, which was necessary given the ethical concerns of conducting a randomized clinical trial, and limited data set available, there may be additional unmeasured confounders that we were not able to control for, such as diagnosis of comorbid mental or substance use–related health conditions, criminogenic risk, and the cause of restrictive housing that may increase risk of mortality and reincarceration. However, we used surrogate measures for these factors including time-varying (at each incarceration period) in-prison mental health and substance use screenings, treatments received, length of each incarceration period, and drug-related or violence-related convictions for each incarceration. We also conducted sensitivity analyses that underscored the robustness of the study results. Despite these limitations, this study aids in identification of a group of individuals at high risk on whom public health interventions could focus, including those related to the current overdose epidemic.^1,2,3^ Future research should build on our findings and more clearly identify the pathways via which restrictive housing affects mortality after release, which will lead to additional interventions for prevention of mortality after release.

## Conclusions

Restrictive housing has been the topic of much policy debate in recent years, during which research has begun to uncover the harms of prolonged exposure. Our results go a step further than other research that we are aware of and highlight the association of restrictive housing with mortality after release. Specifically, our results demonstrate that restrictive housing is associated with a higher likelihood of reincarceration and all-cause mortality, including deaths related to opioid overdose, suicide, and homicide. Importantly, repeated placements and being in restrictive housing for more than 14 days, the threshold of what constitutes torture according to the Mandela Rules, may further exacerbate risk. These findings contribute significantly to the growing body of literature about restrictive housing, suggesting a need to contemplate alternatives to its use and flagging restrictive housing as an important risk factor that must be considered during discharge planning and in the postrelease context for public health systems.

## Notes

Supplement. **eTable. **Association Between Percent-Time Spent in Restrictive Housing During Incarceration and 1-Year Mortality and Reincarceration After Release in North Carolina, 2000-2016
