# Supplementary material for: Association of Restrictive Housing During Incarceration With Mortality After Release
Source: JAMA Netw Open. 2019 Oct 4;2(10):e1912516. doi: 10.1001/jamanetworkopen.2019.12516 (PMC6784785; doi:10.1001/jamanetworkopen.2019.12516)
Supplement: Supplement. — eTable. Association Between Percent-Time Spent in Restrictive Housing During Incarceration and 1-Year Mortality and Reincarceration After Release in North Carolina, 2000-2016 [file jamanetwopen-2-e1912516-s001.pdf]

Supplementary Online Content

Brinkley-Rubinstein L, Sivaraman J, Rosen DL, et al. Association of restrictive housing during incarceration with mortality after release. *JAMA Netw Open.* 2019;2(10):e1912516. doi:10.1001/jamanetworkopen.2019.12516

eTable. Association Between Percent-Time Spent in Restrictive Housing During Incarceration and 1-Year Mortality and Reincarceration After Release in North Carolina, 2000-2016

This supplementary material has been provided by the authors to give readers additional information about their work.

© 2019 Brinkley-Rubinstein L et al. *JAMA Network Open.*

eTable. Association Between Percent-Time Spent in Restrictive Housing During Incarceration and 1-Year Mortality and Reincarceration After Release in North Carolina, 2000-2016

Percentage of incarceration time spent in restrictive housing	Deaths/ re-incarcerations	Person-years	Adjusted[a] Hazard Ratios (95% CI)
**No time (Referent)**	1,557	236,433	1
**>0-5% time**	343	50,310	1.16 (0.94, 1.42)
**>5-10% time**	128	19101	1.25 (0.92, 1.70)
**>10% time**	376	48219	1.41 (1.20, 1.66)
**No time (Referent)**	36,751	236,433	1
**>0-5% time**	5,598	50,310	1.05 (1.00, 1.11)
**>5-10% time**	3120	19101	1.25 (1.18, 1.32)
**>10% time**	13839	48219	1.79 (1.74, 1.84)
**No time (Referent)**	227	236,433	1
**>0-5% time**	39	50,310	1.27 (0.70, 2.29)
**>5-10% time**	20	19101	1.15 (0.66, 2.00)
**>10% time**	46	48219	1.23 (0.80, 1.89)
**No time (Referent)**	230	236,433	1
**>0-5% time**	60	50,310	1.18 (0.81, 1.72)
**>5-10% time**	34	19101	1.50 (0.91, 2.50)
**>10% time**	113	48219	1.75 (1.35, 2.28)
**No time (Referent)**	86	236,433	1
**>0-5% time**	20	50,310	2.34 (1.17, 4.70)
**>5-10% time**	7	19101	0.70 (0.31, 1.58)
**>10% time**	27	48219	2.33 (1.31, 4.14)

a- adjusted for age, gender, race, prior incarcerations, time in incarceration, violent convictions, drug-related convictions, mental health screening recommendation, and mental health treatment receipt.

© 2019 Brinkley-Rubinstein L et al. *JAMA Network Open.*
